# Molecular Imaging of Striatal Dopaminergic Neuronal Loss and the Neurovascular Unit in Parkinson Disease

**DOI:** 10.3389/fnins.2020.528809

**Published:** 2020-09-18

**Authors:** Jana Ivanidze, Myrto Skafida, Sneha Pandya, Dylon Patel, Joseph R. Osborne, Ashish Raj, Ajay Gupta, Claire Henchcliffe, Jonathan P. Dyke

**Affiliations:** ^1^Department of Radiology, Weill Cornell Medicine, Cornell University, New York, NY, United States; ^2^Department of Neurology, Weill Cornell Medicine, Cornell University, New York, NY, United States

**Keywords:** molecular imaging, neurovascular unit, PE2I PET, ASL “arterial spin labeling”, Parkinson disease

## Abstract

Parkinson disease (PD) is the second most common neurodegenerative disorder, characterized by loss of nigrostriatal dopaminergic neurons. Impairment of the neurovascular unit (NVU) has been hypothesized to play a critical role in early PD pathophysiology, and to precede neurodegenerative mechanisms. [C-11]-PE2I (*N*-(3-iodoprop-2E-enyl)-2b-carbomethoxy-3b-(4-methyl-phenyl)nortropane) (PE2I) is a PET radiotracer targeting neuronal dopamine transporters (DaT) with high specificity, allowing for highly accurate and specific DaT quantification. We investigated NVU integrity using arterial spin labeling (ASL) MRI in a prospective cohort of 26 patients with PD, and correlated our findings with analysis of striatal DaT density using PE2I PET in a subcohort of 17 patients. Analysis was performed in FreeSurfer to obtain rCBF and mean standardized regional PET avidity. Pearson correlations and Mann–Whitney tests were performed. Significantly lower mean normalized striatal PE2I SUV values were seen in multiple regions in patients with greater disease duration (*p* < 0.05). PET uptake in the putamen correlated with disease duration independent of patient age. Stratifying patients based on Montreal Cognitive Assessment (MoCA) scores (stratified into ≥ 27 vs. < 27), there was statistically significantly lower PE2I PET avidity in the higher MoCA score group in both more and less affected sides of the caudate, putamen and pallidum (*p* < 0.05). A moderate negative correlation between MDS-UPDRS part 3 (motor) “off” and rCBF values was also seen in the L and R cerebellum WM (*r* = −0.43 and −0.47, *p* < 0.05). A statistically significant negative correlation was found between dominant hand pegboard test results and rCBF in the less affected pallidum (*r* = −0.41; *p* = 0.046). A statistically significant negative correlation of ASL MRI with [11C]-PE2I PET was also found (*r* = −0.53 to −0.58; *p*-value 0.017–0.033) between left cerebral WM rCBF and more and less affected striatal PET regions. Our ROI-based analyses suggest that longer disease duration is associated with lower rCBF and lower PE2I mean SUV, implying greater NVU dysfunction and dopaminergic neuronal loss, respectively. Combined ASL MRI and PE2I PET imaging could inform future prospective clinical trials providing an improved mechanistic understanding of the disease, laying the foundation for the development of early disease biomarkers and potential therapeutic targets.

## Introduction

Parkinson disease (PD) is the second most common neurodegenerative disorder and the most common movement disorder. Traditionally, PD is still diagnosed on the basis of motor symptoms and signs of resting tremor, bradykinesia and rigidity. There are many well-validated and highly studied clinical batteries and rating scales available to quantitate motor features, such as the Movement Disorders Society Unified Parkinson’s Disease Rating Scale (MDS-UPDRS) – Part III “Motor Symptoms off” (Martinez-Martin et al., 2015), or the 9-hole pegboard testing to assess motor dexterity (Proud et al., 2019). However, these classic motor symptoms of PD often present later in the course of the disease, becoming apparent when almost 50–80% of dopaminergic neurons has been lost (Simon et al., 2020). Thus, there is a strong need to identify early disease biomarkers that may allow earlier diagnosis. Furthermore, non-motor features of PD may be as, or more, important. For example mild cognitive impairment (MCI) and dementia are well-recognized as important contributing factors to disease morbidity (Reid et al., 2011). The Montreal Cognitive Assessment (MoCA) score offers a comprehensive assessment of executive cognitive function and is increasingly used as a screening tool in PD (Chou et al., 2014). Criteria for diagnosis of cognitive impairment in PD have been established delineating the importance of early diagnosis of cognitive decline before progression to dementia (Litvan et al., 2012). However, there remains a critical need for biomarkers capable of detecting and tracking changes in PD in the anatomical and network correlates of such important non-motor features.

It is widely accepted that the pathophysiology of PD is related to selective neurodegeneration and loss of dopaminergic neurons projecting from the substantia nigra to the striatum. Earlier pathophysiologic mechanisms that may precede nigrostriatal neuronal loss are the subject of current research. Neuroinflammation with resulting alpha-synuclein accumulation is a key pathophysiological event in PD, and is closely associated with impaired blood-brain-barrier (BBB) permeability (Codolo et al., 2013; Sarkar et al., 2014). Dysfunction of the neurovascular unit (NVU), which is composed of endothelial cells, pericytes, and parenchymal cells (Pelizzari et al., 2019b), correlates with increased BBB permeability and microvascular dysfunction, and has been hypothesized to play a critical role in PD pathophysiology. Cerebral microvascular dysfunction appears to contribute to dopaminergic neuronal loss in PD (Barcia et al., 2004).

Arterial spin labeling (ASL) is a non-contrast MRI technique which allows assessment of cerebral perfusion and is based on the principle of magnetically labeling protons in arterial blood prior to their entry into the tissue of interest (Haller et al., 2016). This provides an advantage over dynamic contrast-enhanced magnetic resonance imaging (DCE-MRI), the latter necessitating the intravenous administration of gadolinium-based contrast agent (GBCA), which is associated with inherent risks (Gulani et al., 2017). ASL allows assessment of relative cerebral blood flow (rCBF) and can quantify NVU dysfunction.

To investigate neuronal dopamine transporter density in PD, [11C] *N*-(3-iodoprop-2E-enyl)-2b-carbomethoxy-3b-(4-methyl-phenyl)nortropane (PE2I) has been increasingly used. PE2I is a PET radiotracer which selectively binds neuronal dopamine transporters, allowing non-invasive *in vivo* quantitation of neuronal dopamine transporter density. The purpose of our study was to investigate dopaminergic neuronal loss and NVU dysfunction in a prospective clinical cohort of patients with PD, using [11C]-PE2I PET and ASL MRI, respectively, as well as to correlate advanced imaging findings with clinical and demographic characteristics, specifically metrics assessing motor symptoms (MDS-UPRDS scores, 9-hole pegboard testing) and cognitive impairment (MoCA).

## Materials and Methods

### Study Population

Twenty six subjects were prospectively enrolled in this institutional review board-approved study. Informed consent was obtained at enrollment from all the participants. Inclusion criteria were PD clinical diagnosis of 3 to 12 years of duration from onset of symptoms, 30 to 70 years-old at time of enrollment, well-established response to one or more dopaminergic agents and/or amantadine, absence of disabling dyskinesias, and PD Hoehn & Yahr stage 2–3, and absence of a clinical diagnosis of dementia. Exclusion criteria were symptoms or signs suggestive of a Parkinson’s plus diagnosis, receiving dopamine receptor blocking agents such as neuroleptics, treatment with acetylcholinesterase inhibitors, a history of brain surgery, history of cancer or other significant medical disease such as autoimmune disorders or others within the past 5 years, any major psychiatric condition and any history of other serious neurological disorders such as a clinically significant stroke, brain tumor, hydrocephalus, epilepsy, other neurodegenerative disorders, encephalitis or repeated head traumas. All enrolled patients were evaluated by a movement disorder specialist at the time of enrollment, with clinical assessment including evaluation of motor, sensory, cognitive and overall disease scores. Clinical chart review was performed by trained research personnel with documentation of duration of disease, symptoms, and medications. MRI and PET/CT studies were performed shortly after enrollment in the study, as discussed below.

### MRI Acquisition

All subjects underwent ASL MRI. All MRI data was acquired on a 3.0 Tesla Siemens Prisma MRI scanner using a 32-channel head resonator. The 3D T_1_-Weighted scan was acquired with 1.0 mm x 1.0 mm x 1.0 mm isotropic resolution using a sagittal MPRAGE sequence with a TR/TE/TI of 1800 ms/2.25 ms/900 ms respectively. The ASL sequence was a 3D pseudocontinuous sequence (J. J. Wang, USC) (Wang et al., 2005). ASL acquisition parameters included a 2000 ms labeling time, a 800 ms post-labeling delay and a 2.5 mm x 2.5 mm x 2.5 mm resolution with 60 slices.

### PET/CT Acquisition

A subcohort of 17 patients underwent [11C]-PE2I PET/CT (performed on the same day as the MRI examination). [11C]-PE2I PET/CT data was acquired on a 64 slice Siemens Biograph mCT scanner. The [11C]-PE2I static images were summed from 30 to 60 min after injection. [11C]-PE2I targets neuronal dopamine transporters (Jakobson et al., 2018). The [11C] PE2I dose administered was 384.1 MBq +/− 51.8 MBq (10.38 ± 1.40 mCi). Resolution was 1.0 mm x 1.0 mm x 2.0 mm, with a 40 cm field of view. The PET images were acquired immediately after the radiotracer injection.

### MRI Processing

**FIGURE 1:**
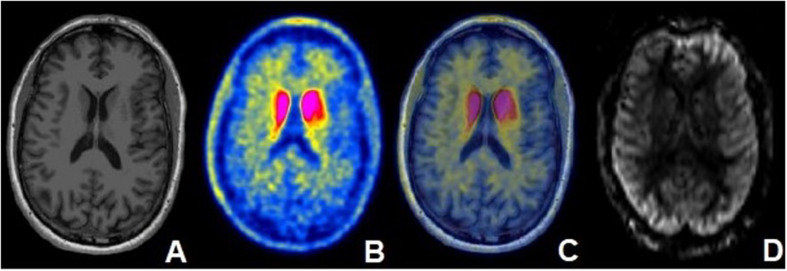
Fifty five year old male with PD diagnosed 5 years ago with moderate disease based on MDS-UPDRS part 3 (motor) “off” score and associated mild cognitive impairment. **(A)** Axial T1-weighted MRI demonstrating no structural abnormalities. **(B)** Axial [11C]-PE2I PET image and **(C)** fused axial [11C]-PE2I PET/MR image demonstrating increased PE2I avidity in the bilateral caudate nuclei. **(D)** rCBF map obtained in the same patient at the same level.

The ASL rCBF data was processed using the MATLAB based ASLtbx (Wang et al., 2008). rCBF images were registered to the 3D T_1_-Weighted data by aligning the proton density (PD) image acquired with the same sequence using the FLIRT package within FSL (FMRIB’s Linear Image Registration Tool; Oxford, United Kingdom) (Jenkinson and Smith, 2001; Jenkinson et al., 2002). The 3D T_1_-Weighted MRI data was analyzed utilizing the FreeSurfer 6.0 image analysis suite^1^ (Fischl et al., 2002; Desikan et al., 2006). Analyzed regions comprised the bilateral striatum (caudate, globus pallidus, putamen) for ASL and [11C]-PE2I PET data. Stratification was performed based on more versus less severely clinically affected side as documented in the standardized clinical neurological examination. Since basal ganglia control contralateral motor function, the opposite side of the one considered more affected clinically was used as the more affected region for imaging purposes. Additionally, the bilateral supratentorial white matter, and bilateral cerebellum was evaluated for ASL analysis for the 26 patients with available ASL data. Axial T_1_ weighted MRI and an rCBF map from a representative patient is presented in Figures 1A,D.

### PET/CT Processing and Co-registration With MRI

The [11C]-PE2I PET data were aligned to the 3D T_1_-Weighted MRI using the FLIRT package in FSL as previously described. Analysis was performed with FreeSurfer 6.0 derived quantitative cortical and subcortical segmentation of structures. [11C]-PE2I PET values were measured for each subregion defined within FreeSurfer. SUV values for the same regions used in our ASL analysis were determined for a subcohort of 17 patients. An SUV for the occipital lobe was generated by combining values from the cuneus, lateral occipital, lingual and pericalcarine regions based on previously published methodology (Desikan et al., 2006). Each target SUV was normalized to this combined occipital SUV. PET findings from a representative patient are presented in Figure 1.

### Subgroup Stratification and Statistical Analysis

**FIGURE 2:**
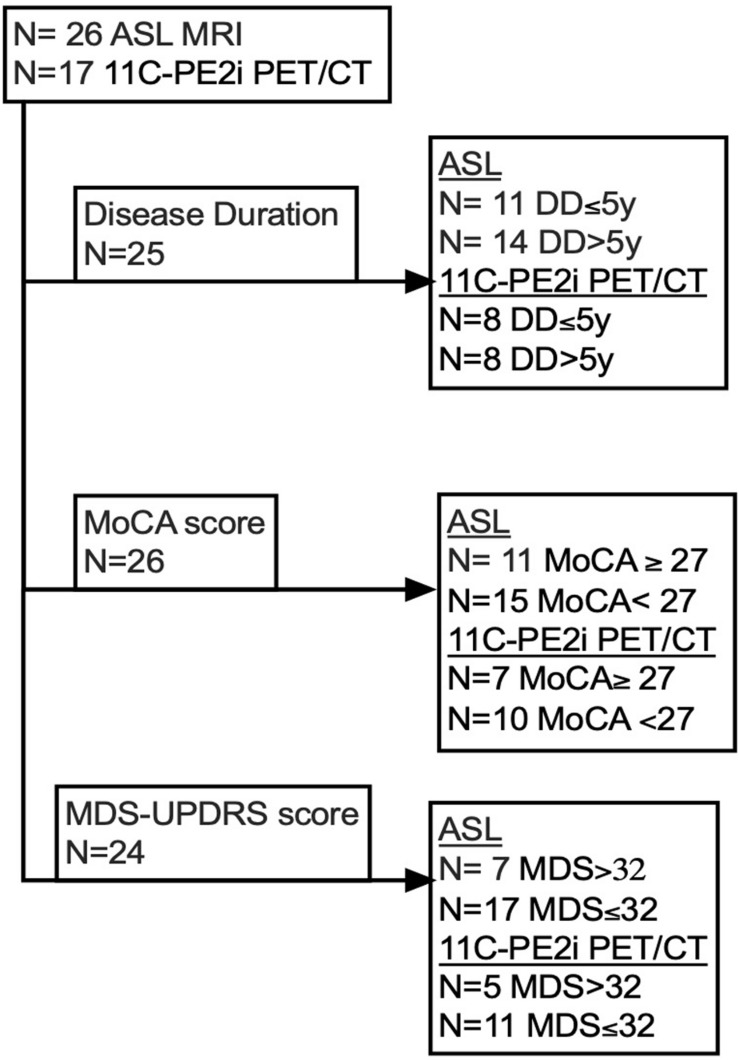
Stratification of the study cohort into subgroups based on clinical characteristics.

Statistical analysis was performed utilizing Graph-Pad Prism version 8. Patients were stratified into the following subgroups based on clinical characteristics. Stratification by duration of disease was based upon a cut-off of 5 years (less than 5 years versus greater than 5 years). Stratification by MoCA and MDS-UPDRS scores was based on previously published data suggesting a cut-off MoCA score of 26 between cognitively normal and cognitively abnormal individuals (Chou et al., 2014), and a cut-off MDS-UPDRS score of 32 for differentiating between mild and moderate disease (Martinez-Martin et al., 2015). We stratified our cohort into subgroups based on clinically meaningful criteria. The final number of patients for each analyzed subgroup is presented in Figure 2. Of note, final numbers for each subgroup depended on available data and thus differed among subgroups, since all 26 patients underwent ASL MRI and a subset of 17 patients underwent PE2I PET. Furthermore, some of the clinical information was not available or clinical tests were not performed for some of the patients. Mann– Whitney *U*-tests were performed to evaluate for differences between subgroups. Correction for multiple comparisons was accomplished using the Benjamini–Hochberg procedure with a false discovery rate (FDR) of 0.1. To investigate the relationship between ASL MRI and [11C]-PE2I -PET data as well as the relationship between clinical scores and imaging data, Spearman correlation analysis was performed. Additionally, a multiple linear regression analysis was performed comparing regional [11C]-PE2I PET uptake normalized to the occipital region with both subject age and disease duration as covariates.

## Results

### Clinical and Demographic Characteristics of the Study Population

Subjects in our cohort had a mean age of 59.5 (range: 44–70, *SD* = ± 5.9) years with mean duration of disease of 6.6 (range: 1–13, *SD* = ± 3.5) years. The mean MDS-UPDRS part 3 (motor) “off” score was 28.7 (range: 12–54, *SD* = ± 11.5) and the mean MoCA score was 25.9 (range: 21–30, *SD* = ± 2.2). With regard to clinical symptoms, 13 (50%) patients had more pronounced symptoms on the left side and 13 (50%) patients had more pronounced symptoms on the right side. 85% (22/26) patients were undergoing treatment with levodopa-containing medications, the majority in combination with other medications, with mean LEDD was 749 mg (*SD* = ± 483). Demographic and clinical characteristics of the subjects enrolled in this study are presented in Table 1.

**TABLE 1:** Clinical and demographic characteristics of the study population.

Number of subjects	26 (F/M: 7/19)
Age (years)	59.5 (range: 44–70 *SD* = ± 5.9)
Duration of disease (years)	6.8 (range: 1–13 *SD* = ± 3.5)
Side of symptom predilection	13 (50%) right; 13 (50%) left
MDS-UPDRS part 3 (motor) “off” score	28.7 (range: 12–54 ± 11.5)
MoCA score	25.9 (range: 21–30 ± 2.2)
**Antiparkinsonian medications**	
Levodopa-containing drugs *n* (%)	22/26 (85%)
Dopamine agonists *n* (%)	16/26 (62%)
MAO-B inhibitors *n* (%)	15/26 (57%)
Anticholinergics *n* (%)	1/26 (38%)
Amantadine and antiglutamate *n* (%)	6/26 (23%)
COMT inhibitors *n* (%)	5/26 (19%)
LEDD mean (SD)	749 (± 483)

### Disease Duration

**FIGURE 3:**
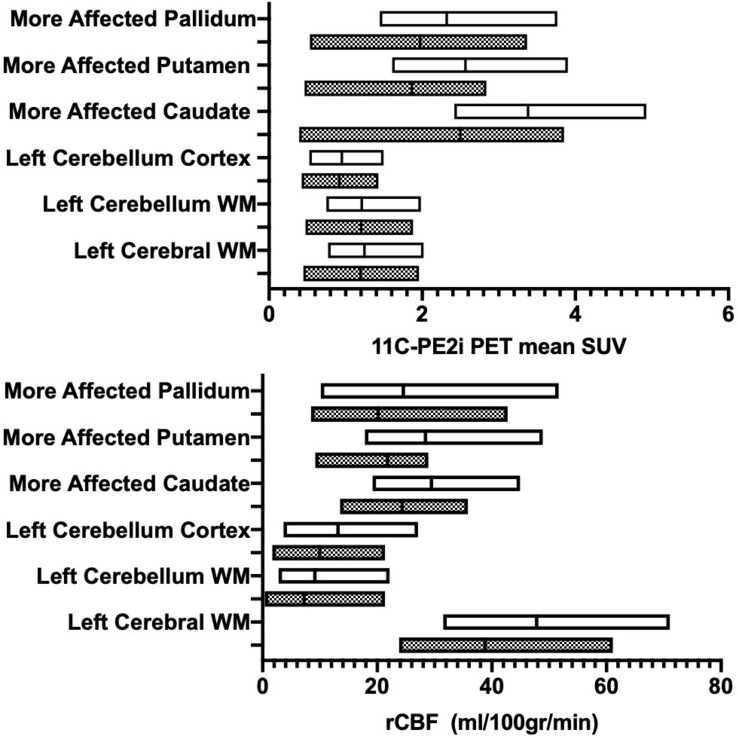
Patients with Parkinson disease (PD) stratified by disease duration (>5 years, gray boxes; ≤5 years, white boxes). Region-specific ASL-based rCBF values (x-axis - **A**) and PE2I-based mean SUV (x-axis- **B**) are provided. Y-axis denotes evaluated brain regions.

Stratification of the cohort based on disease duration resulted in *N* = 11 patients with disease duration of ≤ 5 years (all underwent ASL MRI and a subset of *N* = 8 underwent [11C]-PE2I PET); and *N* = 14 patients with disease duration of > 5 years (all underwent ASL MRI and a subset of *N* = 8 additionally underwent [11C]-PE2I PET). Several regions displayed lower rCBF values with longer disease duration but did not reach significance. A statistically significantly lower PE2I avidity in the group with longer disease duration was shown for multiple regions in Figure 3. The differences between the two groups were more pronounced for [11C]-PE2I SUV in the basal ganglia compared to reference regions (cerebellum cortex and WM, WM). Following Benjamini–Hochberg correction for multiple comparisons, our findings remained significant for all analyzed regions with the exception of left and right cerebellar cortex. rCBF and [11C]-PE2I SUV values for the different regions stratified by disease duration are presented in Table 2.

**TABLE 2:** ASL rCBF and PE2I PET SUV values, stratified by disease duration.

	rCBF values (ml/100 g/min)			PE2I SUV averaged L/R and normalized to occipital		
		
Region of interest	Disease duration ≤5years	Disease duration >5 years	*p*	Disease duration ≤5 years	Disease duration >5 years	*p*
L cerebral WM	41.5	37.7	0.15	0.33	0.29	**0.001**
R cerebral WM	43.6	37.4	0.40	0.33	0.3	**0.049**
L cerebellum WM	6.1	5.7	0.36	0.32	0.3	**0.083**
R cerebellum WM	4.8	4.9	0.46	0.33	0.3	**0.010**
L cerebellum cortex	13.3	9.1	0.24	0.24	0.23	0.130
R cerebellum cortex	11.4	9.0	0.26	0.24	0.2	0.105
More affected caudate	30.3	24.7	0.24	0.94	0.66	**0.001**
Less affected caudate	29.2	26.3	0.76	1.03	0.72	**0.007**
More affected pallidum	22.9	18.5	0.34	0.63	0.51	**0.001**
Less affected pallidum	22.3	20.7	0.60	0.62	0.53	**0.001**
More affected putamen	25.6	24.4	0.10	0.67	0.46	**0.001**
Less affected putamen	26.4	22.6	0.40	0.86	0.54	**0.002**

*Mann–Whitney tests were performed to determine statistical significance between subgroups. Additionally, Benjamini–Hochberg correction for multiple comparisons with an FDR of 0.1 was performed, with *p*-values that remained significant after correction indicated in bold font. R, right; L, left; WM, white matter; p: *p*-value.*

### Montreal Cognitive Assessment

The cutoff to stratify a subject as having MCI/dementia was 27 based on previously published data and therefore 15 patients were considered as having MCI (MoCA scores < 27). Stratification of the cohort resulted in *N* = 15 patients with MoCA scores < 27 (all underwent ASL MRI and a subset of *N* = 10 additionally underwent [11C]-PE2I PET) and *N* = 11 patients with MoCA scores ≥ 27 (all underwent ASL MRI and a subset of *N* = 7 underwent [11C]-PE2I PET). Several regions displayed higher rCBF values in patients with greater MoCA but did not reach significance (Table 3). Furthermore, in the subgroup of patients that had PET data available (*N* = 17) comparing PE2I avidity between the two subgroups, lower [11C]-PE2I avidity in the higher MoCA score group was found with statistically significantly lower [11C]-PE2I SUV values normalized to the occipital lobe in the caudate, pallidum, and putamen (*p* = 0.005-*p* = 0.025) (Table 3). Following Benjamini–Hochberg correction for multiple comparisons, our findings remained significant for all analyzed striatal regions.

**TABLE 3:** ASL rCBF and PE2I PET SUV values, stratified by MoCA score.

	rCBF values (ml/100 g/min)			PE2I SUV averaged L/R and normalized to occipital lobe		
		
Region of interest	MoCA ≥ 27	MoCA < 27	*p*	MoCA ≥ 27	MoCA < 27	*p*
L cerebral WM	45.4	38.7	0.83	0.30	0.33	0.09
R cerebral WM	35.0	43.6	0.60	0.30	0.33	0.09
L cerebellum WM	5.9	5.3	0.46	0.31	0.33	0.11
R cerebellum WM	5.3	4.5	0.60	0.30	0.33	0.09
L cerebellum cortex	11.3	7.9	0.18	0.23	0.24	0.54
R cerebellum cortex	10.5	8.6	0.19	0.23	0.24	0.42
More affected caudate	24.5	28.4	0.68	0.73	0.87	**0.025**
Less affected caudate	24.7	30.2	0.57	0.75	0.97	**0.05**
More affected pallidum	18.6	20.1	0.60	0.49	0.63	**0.005**
Less affected pallidum	23.5	20.1	0.21	0.55	0.81	**0.01**
More affected putamen	24.9	24.5	0.54	0.48	0.66	**0.02**
Less affected putamen	23.1	22.8	0.64	0.55	0.81	**0.01**

*Mann–Whitney tests were performed to determine statistical significance between subgroups. Additionally, Benjamini–Hochberg correction for multiple comparisons with an FDR of 0.1 was performed, with *p*-values that remained significant after correction indicated in bold font. R, right; L, left; WM, white matter.*

When normalized to occipital lobe, there was a moderate negative statistically significant correlation between MoCA scores and normalized SUV in the pallidum on the more affected side (*p* = 0.03) (Table 4).

**TABLE 4:** Correlation between striatal PE2I SUV and MoCA scores.

	SUV normalized to occipital	
	
	*r*	*p*-value
L cerebral WM	−0.23	0.38
R cerebral WM	−0.39	0.12
L cerebellum WM	−0.31	0.22
R cerebellum WM	−0.33	0.20
L cerebellum cortex	−0.12	0.64
R cerebellum cortex	−0.18	0.49
More affected caudate	−0.33	0.20
More affected putamen	−0.45	0.07
More affected pallidum	−0.53	0.03
Less affected caudate	−0.21	0.43
Less affected putamen	−0.33	0.19
Less affected pallidum	−0.37	0.14

*Pearson correlation coefficient r is shown for each striatal sub-region, along with associated *p*-values.*

### MDS VO Part 3

Using Pearson correlation there was a moderate negative correlation between MDS-UPDRS part 3 (motor) “off” and rCBF values in the L and R cerebellum WM (*r* = −0.43 and −0.47, *p* = 0.035 and *p* = 0.021), which may suggest that milder motor symptoms of PD correlate with higher cerebellar rCBF. Patients were additionally stratified based on MDS-UPDRS part 3 (motor) “off” score into groups of mild disease-MDS-UPDRS part 3 (motor) “off” ≤ 32 and moderate disease-MDS-UPDRS part 3 (motor) “off” > 32 (Martinez-Martin et al., 2015). Differences between the two groups were not statistically significant. No patients with an MDS-UPDRS part 3 (motor) “off” 58 (severe disease) were present in our cohort. There was no significant correlation between MDS-UPDRS part 3 (motor) “off” score and [11C]-PE2I SUV values normalized to the occipital lobe.

### Dominant Hand Pegboard Testing

For this analysis, we used all the patients that had available ASL data and Dominant hand Pegboard testing (*N* = 24). A statistically significant, moderate negative correlation was found between Dominant hand Pegboard Test results and rCBF in the less affected pallidum (*r* = −0.41; *p* = 0.046). Dominant hand Pegboard testing did not demonstrate any significant correlations with [11C]-PE2I SUV values in any of the analyzed brain regions. Non-dominant hand Pegboard testing did not demonstrate any significant correlations with rCBF values in any of the analyzed brain regions.

### Combined Imaging

We additionally correlated supratentorial rCBF with striatal [11C]-PE2I PET SUV. For this analysis, we included the 17 patients that had both ASL and [11C]-PE2I PET data. A statistically significant moderate negative correlation was present between left cerebral WM rCBF and more and less affected striatal [11C]-PE2I avidity: more affected caudate (*r* = −0.54, *p* = 0.026), more affected putamen (*r* = −0.53, *p* = 0.031), more affected pallidum (*r* = −0.58, *p* = 0.017) and less affected pallidum (*r* = −0.052, *p* = 0.033). A moderate negative correlation for the left cerebral WM and the less affected caudate (*r* = −0.48) and less affected putamen (*r* = −0.43) did not reach statistical significance (*p* = 0.054 and 0.085, respectively). Analogous analyses for the right cerebral WM demonstrated a similar trend however did not reach statistical significance for either the more or the less affected striatum (*r* = −0.40 to −0.31; *p* = 0.109 to 0.221).

### Effect of Age

**FIGURE 4:**
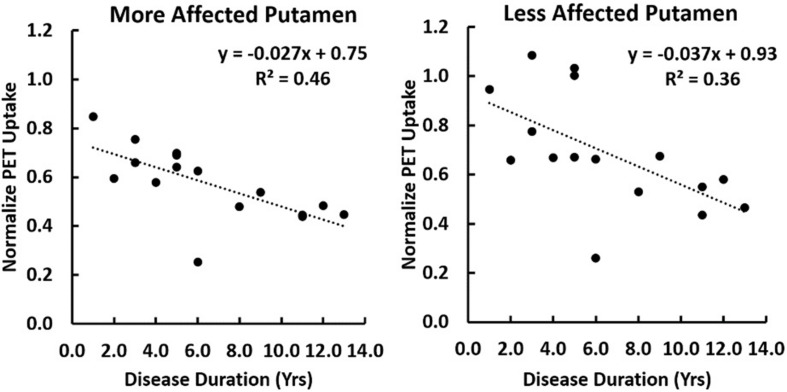
A correlation of [C-11]-PE2I PET uptake normalized to the occipital lobe is shown with disease duration for the putamen lateralized into the more or less affected side. A multiple regression was performed with both subject age as disease duration as covariates. Significant correlation of PET uptake in the putamen with disease duration was found to be *p* = 0.004 and *p* = 0.022 on the more and less affected sides respectively. No significant correlation with subject age was observed.

A multiple linear regression was performed comparing regional [C-11]-PE2I PET uptake normalized to the occipital region with both subject age and disease duration as covariates. Significant correlation of PET uptake in the putamen with disease duration was found to be *p* = 0.004 and *p* = 0.022 on the more and less affected sides respectively. No significant correlation of PET uptake with subject age was observed (Figure 4).

## Discussion

In this study, we combined ASL MRI-based assessment of the NVU with [11C]-PE2I PET-based assessment of striatal dopamine transporter density to assess pathophysiologic mechanisms in a prospective clinical cohort of patients with PD. This approach allowed us to place evaluation of NVU integrity and thereby BBB disruption into a more established clinical context.

At baseline, the BBB has tightly sealed cell–cell contacts involving the capillary endothelial cells and astrocyte endfoot processes, resulting in low transcellular and paracellular permeability. Loss of BBB integrity is associated with decreased relative cerebral blood flow (rCBF) (Montagne et al., 2016), microvascular dysfunction and hemodynamic impairment (Sweeney et al., 2016). BBB permeability is thereby linked to integrity of the NVU (Pelizzari et al., 2019b).

Neuroinflammation with resulting alpha-synuclein accumulation is a hallmark event in PD, and is closely associated with impaired blood-brain-barrier (BBB) permeability and microvascular dysfunction (Zhang et al., 2005; Sarkar et al., 2014; Sweeney et al., 2018), in particular given that certain BBB-targeting metalloproteinases (MMP) can be activated by inflammatory cytokines and chemokines (Germano et al., 2000; Jin et al., 2012). BBB dysfunction in PD has been suggested as a pathophysiologic mechanism based on compelling histopathologic findings including capillary extravasation of cells and proteins, thinning of the capillary endothelial cell layer, and extravascular IgG deposition (Gray and Woulfe, 2015; Pienaar et al., 2015).

Cerebral microvascular dysfunction appears to contribute to dopaminergic neuronal loss in PD (Barcia et al., 2004). Previous work using mouse models of acute, sub-acute and chronic PD demonstrated that area and density of endothelial cells were reduced in regions related to PD pathophysiology such as the substantia nigra and the striatum in all three models, further supporting the importance of microvascular damage in the pathogenesis of PD (Sarkar et al., 2014). The findings of microvascular dysfunction in the substantia nigra have also been suggested by a study comparing number of capillaries, length and diameter of human brain tissue in PD and normal controls (Guan et al., 2013).

NVU dysfunction, associated with a decrease in relative cerebral blood flow (rCBF), has been proposed as an early pathophysiologic mechanism in PD [19], and several ASL-based studies have revealed altered cerebral perfusion patterns in patients with PD compared to normal subjects. [20] ASL-derived differences in rCBF in PD are presently a subject of active investigation, with one study documenting no significant differences in rCBF for any of the evaluated regions (Pelizzari et al., 2019a) while other studies demonstrated decreased striatal rCBF in patients with PD (Wei et al., 2016), concordant with our findings of decreased rCBF in patients with longer disease duration, noting that our study did not include normal subjects for direct comparison. Differences in rCBF distribution have been attributed to BBB dysfunction in PD as well as other neurodegenerative diseases and evaluation of BBB integrity in PD remains the subject of active research. For example, an ASL-MRI-based approach to BBB assessment integrates a diffusion preparation module with pseudo-continuous ASL (pCASL) and a 3D gradient readout (Shao et al., 2019). Appropriate diffusion weighting allows for separation of the tissue and capillary fractions of the ASL signal and in turn allows quantification of capillary permeability of water (kW) between the tissue and intravascular compartments (Shao et al., 2019). Of note, there are currently no diffusion-weighted ASL-based or dynamic-contrast-enhanced (DCE) MRI-based studies in patients with PD.

For evaluation of neuronal dopamine transporter density in PD, to date most imaging studies have used 123I-Iodoflupane (DatSCAN) SPECT, which is in wide clinical use in the work-up of parkinsonian syndrome. [11C]-PE2I PET provides a more accurate and specific quantification of neuronal dopamine transporters in PD compared to DatSCAN SPECT. [11C]-PE2I is a cocaine analog which binds neuronal dopamine transporters with high specificity. While [11C]-PE2I is structurally related to DatSCAN, [11C]-PE2I has the advantage of higher selectivity as it binds neuronal dopamine transporters only, whereas DatSCAN additionally targets serotonin and norepinephrine transporters (Jakobson et al., 2018). [18F] *N*-(3-fluoropropyl)-2β-carboxymethoxy-3β-(4-iodophenyl)nortropane (FP-CIT) has recently been demonstrated to have utility in dopamine transporter imaging in PD and related syndromes, with comparable image quality to [11C]-PE2I (Hong et al., 2018).

Another approach to visualization of dopamine depletion is [F-18]-Fluoro-DOPA (FDOPA) PET. FDOPA is limited in quantifying neuronal dopamine transporters as it illustrates the activity of aromatic amino acid decarboxylase which represents an intraneuronal reaction and is not specific for dopamine. Published data from a direct comparison of F-DOPA and [11C]-PE2I demonstrated greater sensitivity of [11C]-PE2I for the detection of differences in motor symptom severity and progression over time in PD (Li et al., 2018). Studies comparing [11C]-PE2I PET to combined DatSCAN SPECT and 18F-FDG PET further demonstrated superiority of [11C]-PE2I PET (Appel et al., 2015).

To our knowledge, this is the first study investigating the concurrent use of ASL imaging and [11C]-PE2I PET. We found statistically significantly lower striatal PE2I avidity in the sub-cohort of patients with longer disease duration, as well as a trend for lower rCBF values in the same cohort.

The significant negative correlations we identified between MoCA scores and [11C]-PE2I SUV values in the basal ganglia reflect current understanding that cognitive impairment in PD is not, for the most part, directly tied to dopaminergic neuronal loss, as patients with lower MoCA score had higher [11C]-PE2I SUV. Of note, cognitive impairment has been suggested to precede the development of “classic” motor symptoms in PD (Fereshtehnejad et al., 2019), and while our study only incorporates one imaging time point, this may reflect a pathophysiologic basis for our findings and needs to be studied further in future longitudinal studies.

Previously published data have shown that rCBF values in basal ganglia are inversely correlated to motor dysfunction (Wei et al., 2016). Stratification based on MDS-UPDRS score into groups of mild disease-MDS ≤ 32 and moderate disease-MDS-UPDRS > 32 did not reveal a clear trend for rCBF values, although Pearson correlation analysis did demonstrate a weak negative correlation between MDS-UPDRS and cerebellar rCBF values.

Our study has several limitations, most notably small sample size overall as well as small sample size of the various subgroups. Another limitation is lack of direct comparison to non-diseased subjects. Furthermore, our study is cross-sectional and lacks longitudinal follow-up data. However, we demonstrate feasibility of concurrent non-invasive imaging of cerebral perfusion and dopaminergic neuronal loss, and place our findings in the clinical context of motor and cognitive decline in PD.

## Conclusion

Our ROI-based analyses suggest that longer disease duration is associated with lower rCBF and lower striatal [11C]-PE2I avidity, implying greater NVU dysfunction and greater dopaminergic neuronal loss, respectively. Correlative studies of ASL and [11C]-PE2I PET data further suggest that dopaminergic neuronal loss may be independent from cognitive impairment in PD.

Combined perfusion MRI and dopamine transporter targeted PET imaging may inform future prospective clinical trials, thereby providing an improved mechanistic understanding of PD and related neurodegenerative diseases, and laying the foundation for the development of early disease biomarkers and potential therapeutic targets.

## Data Availability Statement

All datasets generated for this study are included in the article/supplementary material.

## Ethics Statement

The studies involving human participants were reviewed and approved by Institutional Review Board – Weill Cornell Medicine. The patients/participants provided their written informed consent to participate in this study.

## Author Contributions

JI conceptualized the project, analyzed the data, and wrote the manuscript. SP collected the PET data. JD collected the MRI data. AR, AG, and CH conceptualized the prospective PD observational study that served as the basis for this project. DP and CH evaluated the subjects and collected clinical and demographic data. All authors contributed to data analysis and manuscript writing.

## Conflict of Interest

The authors declare that the research was conducted in the absence of any commercial or financial relationships that could be construed as a potential conflict of interest.

## Footnotes

1 http://surfer.nmr.mgh.harvard.edu/
